# A case of atypical meningioma presenting spontaneous infarction: the findings of magnetic resonance imaging, including amide proton transfer-chemical exchange saturation transfer imaging

**DOI:** 10.1093/bjrcr/uaae023

**Published:** 2024-07-09

**Authors:** Etsushi Iida, Atsuo Inoue, Masaya Tanabe, Naohiko Kamamura, Katsuyoshi Ito

**Affiliations:** Department of Radiology, Graduate School of Medicine, Yamaguchi University, Ube 755-8505, Japan; Department of Radiology, Graduate School of Medicine, Yamaguchi University, Ube 755-8505, Japan; Department of Radiology, Graduate School of Medicine, Yamaguchi University, Ube 755-8505, Japan; Department of Radiology, Graduate School of Medicine, Yamaguchi University, Ube 755-8505, Japan; Department of Radiology, Graduate School of Medicine, Yamaguchi University, Ube 755-8505, Japan

**Keywords:** atypical meningioma, spontaneous infarction, amide proton transfer-chemical exchange saturation transfer imaging

## Abstract

We report the MRI findings of a patient with an atypical meningioma who presented with spontaneous infarction. A 67-year-old man with histories of recurrent meningioma complained of left ocular protrusion and a subsequent biopsy revealed atypical meningioma. Contrast-enhanced CT showed a uniformly enhancing tumour in the left ethmoid sinus, but MRI 2 days later showed no enhancement on Gd-T1WI and severe diffusion restriction on DWI, indicating spontaneous infarction. APT-CEST imaging showed slight hypointensity in comparison to the normal brain with a mean MTR asymmetry value of 0.48%. Tumour regrowth was confirmed on MRI after 2 months. The recurrent tumour showed moderate diffusion restriction on DWI and hyperintensity with a mean MTR asymmetry value of 2.59% on APT-CEST imaging. The decreased signal on APT-CEST at the time of spontaneous infarction may have been attributed to intratumoral acidosis and loss of viable tumour. APT-CEST imaging is useful for evaluating the intratumoral condition and tumour viability of the infarcted or ischemic tumour.

## Introduction

Meningioma is the most common primary brain tumour in adults and is derived from the meningothelial cells of the arachnoid mater. Atypical and anaplastic meningioma are relatively rare and show a higher recurrence rate and worse prognosis.^1–3^ Spontaneous infarction of meningioma is extremely rare, and few cases have been reported with imaging findings.^4–6^ We report a case of atypical meningioma that presented with spontaneous infarction and subsequent rapid regrowth. We focus on the MRI findings, including diffusion-weighted imaging (DWI) and amide proton transfer-chemical exchange saturation transfer (APT-CEST) imaging. To our knowledge, this is the first case report of APT-CEST imaging for a patient with atypical meningioma who presented with spontaneous infarction.

## Case presentation

A 67-year-old man visited our hospital complaining of left ocular protrusion. He underwent a total resection of olfactory groove meningioma (WHO grade 1) 13 years ago and CyberKnife radiosurgery for 2 recurrences of the cerebral falx and left sphenoid ridge, 11 and 5 years ago. The third recurrence occurred 2 years ago in both the cerebral falx and the left sphenoid ridge; the histopathological diagnosis after total resection was still meningioma (WHO grade 1).

CT revealed a mass with homogenous soft tissue density, measuring 58 × 39 mm, located in the left ethmoid sinus and extending into the left orbit, nasal sinus, left maxillary sinus, and sphenoid sinus. He was admitted on the same day for further examination and treatment. A biopsy from the tumour protruding into the nasal sinus on day 2 of admission revealed atypical meningioma (WHO grade 2) with histological findings of atypical cells with a high N: C ratio, prominent nucleoli, and foci of necrosis.

**Figure 1.**
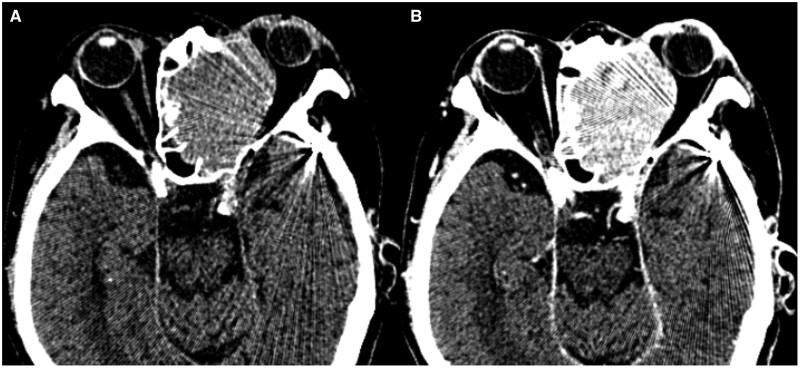
(A, B) Follow-up CT after biopsy on the day 14 of admission. Non-contrast CT shows a homogeneous high-density mass in the left ethmoid sinus extending to the left orbit (A). Contrast-enhanced CT shows homogenous enhancement in the tumour (B).

**Figure 2.**
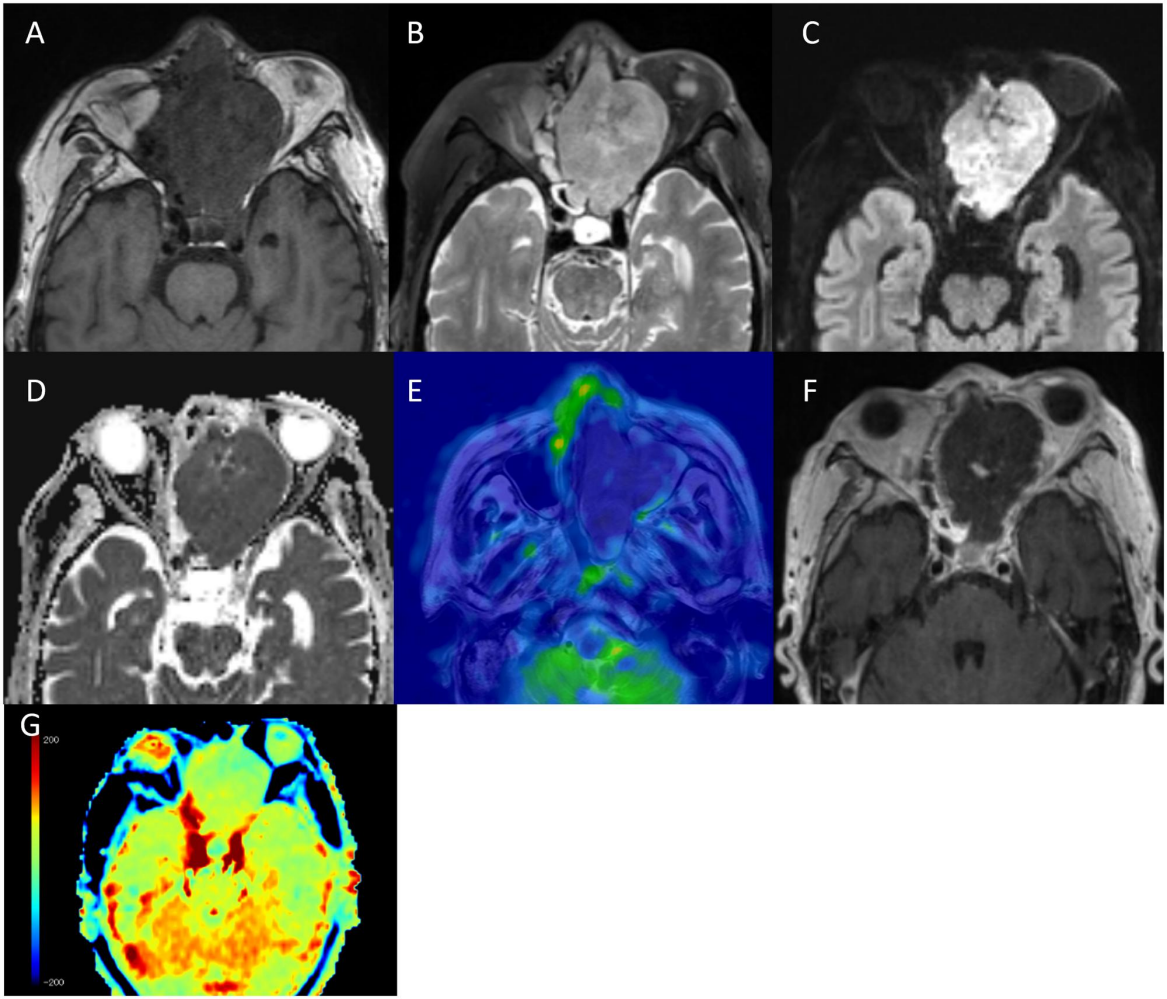
(A-G) MRI on day 16 of admission. The tumour in the left ethmoid sinus shows an isointense signal in comparison to muscle on T1WI (A), a hyperintense signal on T2WI (B), and a hyperintense signal with a low ADC value (mean ADC value 0.72 × 10^−3^ mm^2^/s) on DWI (C, D). Arterial spin label perfusion-weighted imaging (ASL-PWI) shows a hypointense signal in the tumour (E). Gd-T1WI shows an extensive unenhanced area in the tumour (F), suggesting spontaneous infarction of the tumour (E, F). APT-CEST imaging shows a slight hypointense signal in the unenhanced area within the tumour in comparison to normal brain parenchyma (G).

Follow-up CT on day 14 of admission showed uniform enhancement within the tumour (Figure 1). The patient complained of left eye pain and sudden complete blindness on day 15 and then underwent MRI on a 3-Tesla scanner on day 16. Gd-T1WI showed extensive areas of non-enhancement within the tumour (Figure 2F). The tumour was isointense in comparison to muscle on T1WI (Figure 2A) and hyperintense on T2WI (Figure 2B). On DWI, the tumour showed a hyperintense signal with a low ADC value (mean ADC value 0.72 × 10^−3^ mm^2^/s) (Figure 2C and D). An arterial spin label perfusion-weighted image (ASL-PWI) with a post-labelling delay of 2000 ms revealed a hypointense signal consistent with the unenhanced area in the tumour (Figure 2E). The course of these imaging findings on CT and MRI suggests spontaneous infarction of the tumour.

Amide proton transfer (APT)-CEST (magnetization transfer asymmetry [MTRasym] at 3.5 ppm) imaging showed a slight hypointense signal in the unenhanced area within the tumour in comparison to the normal brain parenchyma. The mean MTRasym value was 0.48% and the normalized MTRasym (nMTRasym) value, which was defined as MTRasym (tumour)—MTRasym (normal brain parenchyma), was −0.55% (Figure 2G).

**Figure 3.**
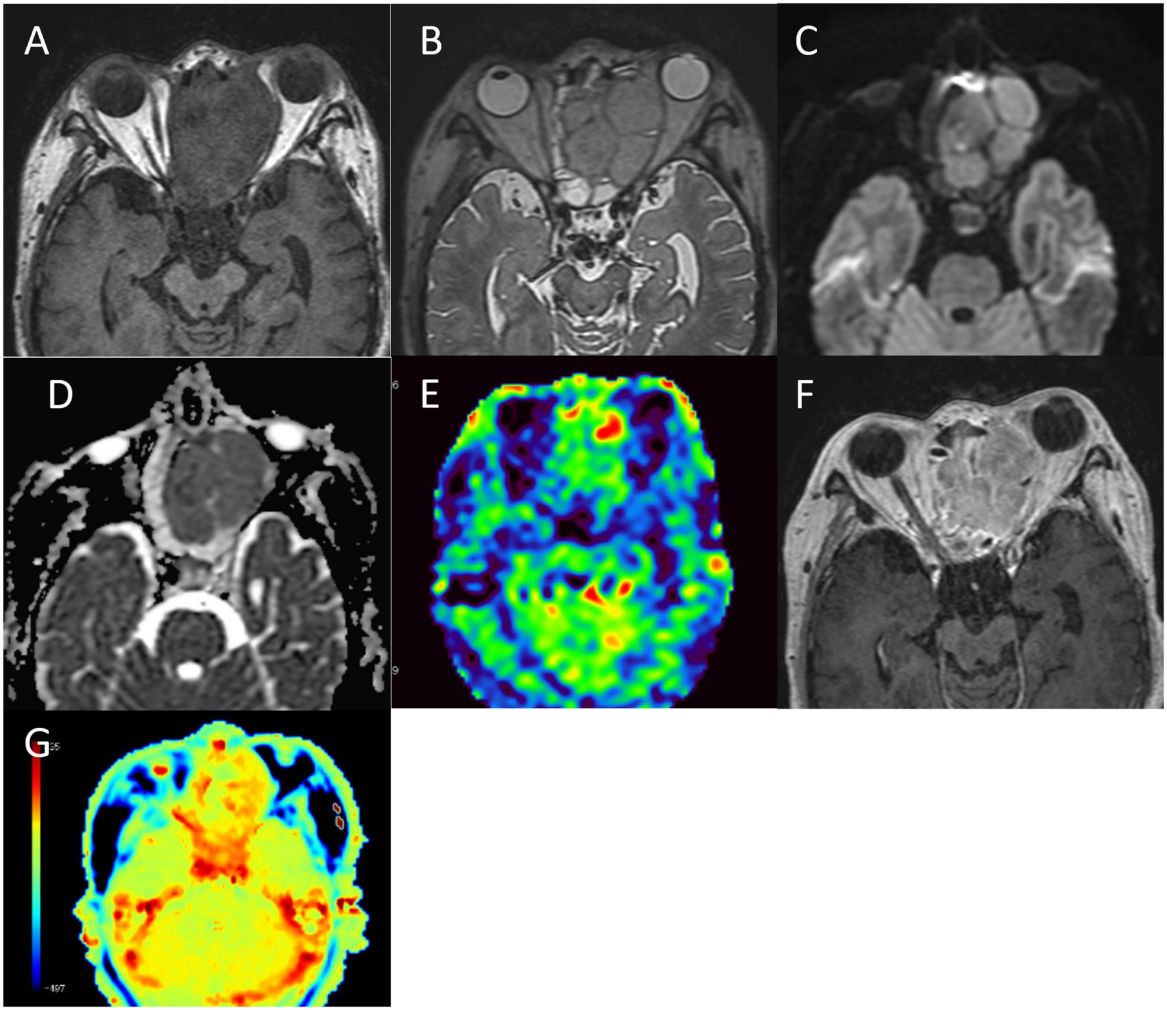
(A-G) MRI three months after admission. MRI shows a recurrent tumour in the left ethmoid sinus with an isointense signal in comparison to muscle on T1WI (A), hyperintense signal on T2WI (B) and a hyperintense signal with a low ADC value (mean ADC value 0.91 × 10^−3^ mm^2^/s) on DWI (C, D). On ASL-PWI, the signal of the tumour is isointense in comparison to normal brain parenchyma (E) and Gd-T1WI shows homogeneous enhancement (F). APT-CEST imaging shows a hyperintense signal in the tumour in comparison to the normal brain parenchyma (G).

At 1 month after admission, MRI showed a decreased signal intensity in the tumour on DWI, with an increased ADC value of 1.23 × 10^−3^ mm^2^/s. New enhancing areas appeared within the tumour on Gd-T1WI 2 months after admission. These enhancing areas showed a hyperintense signal on DWI with a relatively low ADC value (mean ADC value 0.9 × 10^−3^ mm^2^/s). MR spectroscopy (TE = 30 ms) showed marked elevation of the choline peak and moderate elevation of the lipid/lactate peak. These findings indicate tumour regrowth within the necrotic area. The recurrent tumour grew progressively and filled the upper nasal cavity and the ethmoid sinus 3 months after admission. The tumour showed an isointense signal in comparison to muscle on T1WI (Figure 3A) and a hyperintense signal on T2WI (Figure 3B). On DWI, the tumour showed a hyperintense signal with a relatively low ADC value (mean ADC value 0.91 × 10^−3^ mm^2^/s) (Figure 3C and D). ASL-PWI revealed an isointense signal in comparison to normal brain parenchyma (Figure 3E). Gd-T1WI showed homogeneous enhancement (Figure 3F). APT-CEST imaging showed a hyperintense signal in comparison to normal brain parenchyma with a mean MTRasym value of 2.59% and a normalized MTRasym value of 2.33% (Figure 3G).

The patient underwent palliative radiation therapy, and the tumour slightly regressed in 4 months. Then, tumour regrowth and intracranial extension occurred followed by the development of an intracranial abscess adjacent to the tumour, and the patient died approximately 1 year after admission.

## Discussion

Meningioma accounts for 37.6% of CNS tumours. Most meningiomas are benign and are classified as CNS WHO grade 1.^3^ However, meningiomas tend to become more histologically malignant with each recurrence. Malignant transformation occurs in 0.16%-2% of all cases.^1^^,^^2^ Atypical and anaplastic meningiomas are seen in 5%-15% and 1%-2% of all meningiomas, respectively.^2^ Atypical meningioma is an intermediate-grade (CNS WHO grade 2) meningioma that has histopathologic features such as increased mitotic activity, brain invasion, high cellularity, small cells with a high N: C ratio, prominent nucleoli, sheeting, and necrotic foci. The time for malignant transformation ranges from 8 months to 26 years.^1^ Factors of malignant transformation include irradiation, surgical stress, and viral infections. Growth factors such as VEGF and chromosomal mutations are also considered to be implicated.^1^

Spontaneous infarction of meningioma is reported to be rare and seen in the centre of the tumour because the feeding artery supplies the periphery of the tumour and central tissue is more vulnerable to hypoperfusion or hypotension.^4^ Infarcted meningioma shows a hyperintense signal on DWI with a low ADC value in the acute phase, similar to cerebral infarction, while Hall et al reported lack of diffusion restriction at 10 days after the onset of symptoms.^5^^,^^6^ In this case, the tumour was uniformly enhanced on contrast-enhanced CT on day 14 of admission, but only 2 days later, most of the tumour lacked enhancement on Gd-T1WI. Furthermore, the tumour showed severe hypoperfusion on ASL-PWI and ADC values (mean ADC value 0.72 × 10^−3^ mm^2^/s) were lower than those in the regenerative phase (mean ADC value 0.91 × 10^−3^ mm^2^/s). Therefore, we consider that these MRI findings on day 16 of admission indicate a spontaneous acute infarction of atypical meningioma.

Chemical exchange saturation transfer (CEST) imaging is a molecular imaging technique on MRI that allows the visualization of the distribution of trace molecules in the body. CEST imaging indirectly visualizes trace molecules by utilizing the proton exchange phenomenon between protons in trace molecules and bulk water, which is abundant in the body. By applying a saturation pulse at a specific frequency to the protons in trace molecules, the signal intensity of bulk water indirectly decreases due to the proton exchange phenomenon. By observing this signal change, it is possible to confirm the presence or absence of trace molecules. Trace molecules that are detectable by CEST include endogenous biomolecules with amide, amine, and hydroxyl groups, as well as exogenous agents, such as natural D-glucose, which contain exchangeable protons.

APT-CEST imaging visualizes the distribution of the amide protons in mobile proteins and peptides, such as those in the cytoplasm, through chemical exchange between exchangeable amide protons in the protein peptide bonds and tissue water. Togao et al reported the usefulness of APT-CEST imaging in predicting the histological grade of adult diffuse gliomas.^7^ The MTRasym value (@3.5 ppm) in malignant glioma was higher than that in low-grade glioma (4.1 ± 1.0% in grade IV, 3.2 ± 0.9% in grade III, and 2.1 ± 0.4% in grade II). They also showed a positive correlation between the signal intensity on APT-CEST imaging and the Ki-67 labelling index or cell density, indicating that active proliferation of tumour cells is related to a high concentration of mobile proteins and peptides.^7^

Meningioma imaging using APT-CEST has shown that atypical meningioma displays a hyperintense signal in comparison to benign meningioma. The normalized MTRasym values for benign meningiomas and atypical meningiomas were 1.67% and 2.46%, respectively.^8^ The regrowing atypical meningioma in the present case showed a normalized MTRasym value of 2.33%, consistent with the previous report.^8^

APT-CEST imaging is also reported to be useful for evaluating tumour viability of brain tumours.^9^^,^^10^ Ma et al assessed APT-CEST imaging features in patients with malignant gliomas after chemoradiation and reported that the diagnostic performance of APT-CEST imaging for distinguishing true progression from pseudo-progression is good. They found that the MTRasym values (@3.5 ppm) were higher in patients with true progression (2.75% ± 0.42%) than in those with pseudo-progression (1.56% ± 0.42%).^10^

APT CEST imaging is also known to be available for pH imaging based on the amide proton exchange rate and detecting ischaemic tissue acidosis in human stroke patients. Signal intensity decreases in the infarcted area on MTRasym (@3.5 ppm) map due to a decrease in the exchange rate of amide protons.^11^ Thus, in the present case, the low signal intensity (mean MTRasym value 0.48%) on APT-CEST imaging at the time of spontaneous tumour infarction may have been caused by a combination of the low content of mobile protein and peptides due to tumour necrosis and a decrease in pH in the tumour. In contrast, the high signal intensity (MTRasym value of 2.59%) on APT-CEST imaging at the time of progressive regrowth of the tumour is considered to indicate a high content of mobile protein and peptides due to the active proliferation of tumour cells.

In summary, we presented a case of atypical meningioma presenting spontaneous infarction. In the acute phase of tumour infarction, DWI showed diffusion restriction and APT-CEST imaging showed a hypointense signal, probably due to the loss of viable tumour and intratumoral acidosis. In the regrowth phase, APT-CEST imaging showed a hyperintense signal due to the active proliferation of tumour cells. APT-CEST imaging provides information within the tumour that differs from conventional MR sequences.

## Learning points

- Infarcted atypical meningioma in acute phase shows diffusion restriction on DWI and ADC maps.
- Infarcted atypical meningioma shows a hypointense signal on APT-CEST imaging due to the loss of viable tumour and intratumoral acidosis.
- APT-CEST imaging shows a hyperintense signal due to the active proliferation of tumour cells in the regrowth phase.

## References

[1] Ito K , Imagama S, Ando K, et al Intraspinal meningioma with malignant transformation and distant metastasis. Nagoya J Med Sci. 2017;79(1):97-102. 10.18999/nagjms.79.1.97 28303067 PMC5346626

[2] Baek BJ , Shin JM, Lee CK, Lee JH, Lee KH. Atypical primary meningioma in the nasal septum with malignant transformation and distant metastasis. BMC Cancer. 2012;12:275. 10.1186/1471-2407-12-275 22759338 PMC3420263

[3] Ostrom QT , Cioffi G, Gittleman H, et al CBTRUS Statistical Report: Primary Brain and Other Central Nervous System Tumors Diagnosed in the United States in 2012-2016. Neuro Oncol. 2019;21(Suppl 5):V1-V100. 10.1093/neuonc/noz150 31675094 PMC6823730

[4] Lee BH. Rapid progression of central necrosis in a large extra-axial tumor following aneurysmal subarachnoid hemorrhage. Interdisciplinary Neurosurgery. 2020;22:100860. 10.1016/j.inat.2020.100860

[5] Peri N , Lee PH, Anderson MP, Bhadelia RA. Acute infarction of meningioma demonstrated by diffusion-weighted MR imaging. J Neurooncol. 2008;90(3):275-278. 10.1007/s11060-008-9673-7 18726186

[6] Hall J , Wang YY, Smith P, Sutherland T. Spontaneous infarction within a meningioma with negative DWI: an imaging pattern in patients with acute neurological deterioration. BJR Case Rep. 2015;1(2):20150039. 10.1259/bjrcr.20150039 30363176 PMC6159131

[7] Togao O , Yoshiura T, Keupp J, et al Amide proton transfer imaging of adult diffuse gliomas: Correlation with histopathological grades. Neuro Oncol. 2014;16(3):441-448. 10.1093/neuonc/not158 24305718 PMC3922507

[8] Joo B , Han K, Choi YS, et al Amide proton transfer imaging for differentiation of benign and atypical meningiomas. Eur Radiol. 2018;28(1):331-339. 10.1007/s00330-017-4962-1 28687916 PMC5746026

[9] Jiang S , Eberhart CG, Lim M, et al Identifying recurrent malignant glioma after treatment using amide proton transfer-weighted MR imaging: a validation study with image-guided stereotactic biopsy. Clin Cancer Res. 2019;25(2):552-561. 10.1158/1078-0432.CCR-18-1233 30366937 PMC6335169

[10] Ma B , Blakeley JO, Hong X, et al Applying amide proton transfer-weighted MRI to distinguish pseudoprogression from true progression in malignant gliomas. J Magn Reson Imaging. 2016;44(2):456-462. 10.1002/jmri.25159 26788865 PMC4946988

[11] Heo HY , Tee YK, Harston G, Leigh R, Chappell MA. Amide proton transfer imaging in stroke. NMR Biomed. 2023;36(6):e4734. 10.1002/nbm.4734 35322482 PMC9761584

